# Spontaneous Hemoperitoneum From a Ruptured Ovarian Cyst as the Initial Presentation of Aplastic Anemia in an Adolescent Female Patient: A Case Report

**DOI:** 10.7759/cureus.97225

**Published:** 2025-11-19

**Authors:** Mohammad Damseh, Joana Almeida, Charles Gigi, Ajesh Sankar, Mona Fawzy

**Affiliations:** 1 Department of Obstetrics and Gynaecology, Barnsley Hospital NHS Foundation Trust, Barnsley, GBR

**Keywords:** aplastic anemia, hemoperitoneum, hemorrhagic ovarian cyst, immunosuppresive therapy, ovarian suppression

## Abstract

Severe spontaneous hemoperitoneum resulting from a ruptured ovarian cyst is an uncommon but potentially life-threatening condition. Its occurrence as the initial presentation of underlying aplastic anemia is even more unusual, with only a handful of cases reported in the literature. We present the case of a 17-year-old female patient whose first manifestation of undiagnosed aplastic anemia was significant intra-abdominal bleeding from a ruptured ovarian cyst, requiring emergency laparoscopic intervention. Subsequent investigations confirmed aplastic anemia of unknown etiology. The patient is currently receiving immunosuppressive therapy while awaiting an allogeneic bone marrow transplant. In the interim, ovulation suppression has been initiated to minimize the risk of recurrent hemorrhage.

## Introduction

Aplastic anemia is a rare but serious bone marrow failure syndrome that can present insidiously or with acute complications. Its coexistence with paroxysmal nocturnal hemoglobinuria (PNH) clones is well-documented, though often subclinical [1]. We present the case of a previously healthy 17-year-old female patient who initially presented with acute abdominal pain due to a hemorrhagic ovarian cyst and was subsequently diagnosed with aplastic anemia with a minor PNH clone. Her case highlights the diagnostic complexity of pancytopenia in the context of gynecological emergencies and the challenges of managing ongoing bleeding risks, transfusion dependence, and fertility considerations in adolescent patients.

## Case presentation

**Figure 1:**
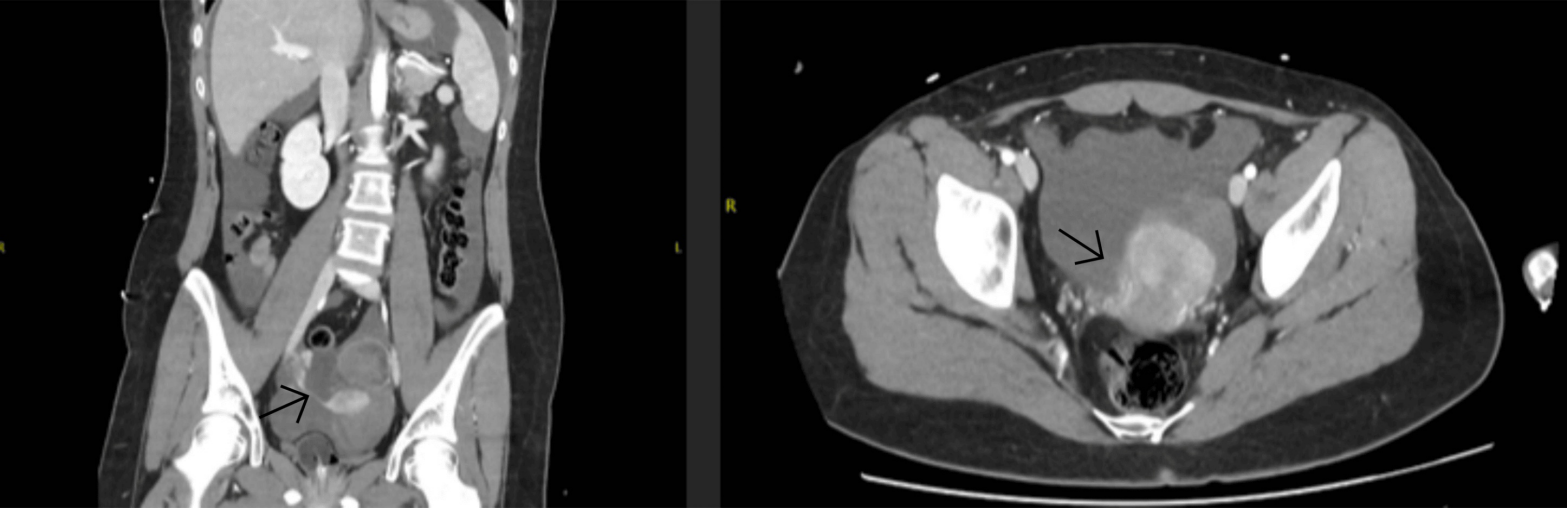
A CT of the abdomen and pelvis showing bleeding from the left ovary/ adnexa

A 17-year-old previously healthy female patient presented on 25 October 2024 to the emergency department with a one-day history of worsening right iliac fossa pain, associated with pallor and dizziness but no nausea, vomiting, or changes in bowel or urinary habits. She had never been sexually active, with menarche at age 11 and a history of irregular but not heavy menstrual periods. There was no significant family history. On examination, she was tachycardic (heart rate (HR): 112 bpm), normotensive (blood pressure (BP): 131/81 mmHg), and afebrile (temperature: 37.1°C), with signs of an acute abdomen. Per rectal exam, a full, tender pouch of Douglas was revealed; vaginal examination was limited by an intact hymen. Initial blood investigations (Table 1) showed pancytopenia. Coagulation was unremarkable. Total bilirubin was mildly elevated. Beta-human chorionic gonadotropin (β-HCG) was negative. A CT of the abdomen revealed moderate hemoperitoneum, an enlarged heterogeneous left ovary, and high-density material in the left adnexa suggestive of acute clot (Figure 1).

**Table 1:** Initial blood investigations

Parameter	Result	Unit	Reference range
Hemoglobin	55	g/L	117-157
White blood cells	5.2	10⁹/L	4.0-10.0
Platelets	12	10⁹/L	170-400
Red blood cell (RBC)	1.35	10⁹/L	4.00-5.10
Hematocrit	0.15	L/L	0.35-0.46
Mean corpuscular volume (MCV)	116	fL	82.0-100.0
Mean corpuscular hemoglobin (MCH)	40.8	pg	27.0-32.5
Mean corpuscular hemoglobin concentration (MCHC)	351	g/L	316-165
Red cell distribution width (RDW)	17		12.2-15.4
Neutrophils	1.4	10⁹/L	1.7-6.6
Serum total bilirubin	36	µmol/L	0-20.0
Alanine aminotransferase (ALT)	10	U/L	10-49
Amylase	<20	U/L	30-118
International normalized ratio (INR)	1.1		

**Figure 2:**
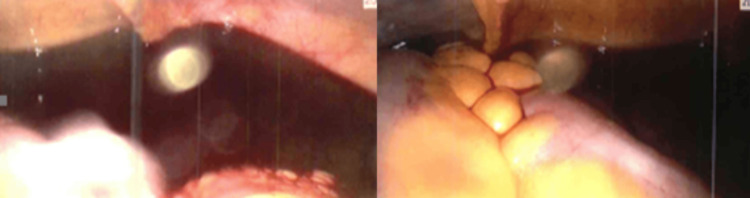
A hemoperitoneum was identified during laparoscopy.

Hematology consultant input was sought, and she received one unit of RBC and platelet transfusion, which led to partial improvement (hemoglobin 81 g/L, platelet 64 ×10⁹/L). Further investigations (Table 2) revealed a reticulocyte count of 34, lactate dehydrogenase was low at 80 U/L, vitamin B12 was low-normal at 192 ng/L (treated later with replacement), and folate was normal. Diagnostic laparoscopy (Figure 2) confirmed a ruptured and actively bleeding left ovarian cyst with 400 mL hemoperitoneum; washout and diathermy were performed. The right ovary and both fallopian tubes appeared normal.

**Table 2:** Further blood investigations

Parameter	Result	Unit	Reference range
Reticulocyte count	34	×10⁹/L	25-110
Lactate dehydrogenase (LDH)	80	U/L	120-246
Vitamin B12	192	ng/L	211-911
Folate	5.9	µg/L	>5.4

She was admitted to the ICU postoperatively and discharged six days later with ongoing cytopenias (hemoglobin 93 g/L, platelets 25×10⁹/L, WBC 3.1×10⁹/L). The blood film showed anisopoikilocytosis, teardrop cells, macrocytosis, and polychromasia. Follow-up revealed persistent anemia and thrombocytopenia requiring multiple outpatient transfusions. She remained transfusion-dependent, with weekly blood monitoring and transfusions as clinically indicated. Viral serologies (Epstein-Barr virus, cytomegalovirus, and parvovirus) were IgG positive but IgM negative; erythropoietin was markedly elevated at >750 IU/L (reference 4.3-29.0 IU/L), and β2-microglobulin was slightly low at 1.1 mg/L (reference 1.2-2.4 mg/L). ANA was negative. Flow cytometry confirmed a minor PNH clone: neutrophils 3.16%, monocytes 2.54%, and red cells 0.28% (type II 0.14%, type III 0.14%) with absent or reduced expression of GPI-linked antigens (CD24, CD10, and CD59). Bone marrow biopsy showed a severely hypocellular marrow with near-complete absence of hematopoietic cells, consistent with aplastic anemia. No evidence of dysplasia, fibrosis, or malignancy was seen. The myeloid next-generation sequencing (NGS) panel revealed no pathogenic variants. Lymphocyte analysis showed a T-cell-dominant marrow (CD3+ 82%) and polyclonal B-cells (6%). She was diagnosed with aplastic anemia with a subclinical PNH clone and commenced on immunosuppressive therapy with anti-thymocyte globulin, cyclosporin, and eltrombopag. An allogeneic bone marrow transplant from a matched sibling donor was scheduled for September 2025. The patient was informed about fertility preservation options, but expressed that this was not a current priority.

**Figure 3:**
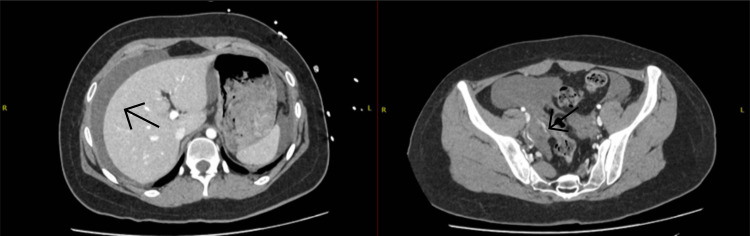
A CT of the abdomen and pelvis shows hemoperitoneum and an area of concern: ongoing bleeding in the right ovary.

She re-presented three months later (19^th^ January 2025) with epigastric and periumbilical pain. A CT confirmed active hemoperitoneum due to right ovarian bleeding (Figure 3).

**Figure 4:**
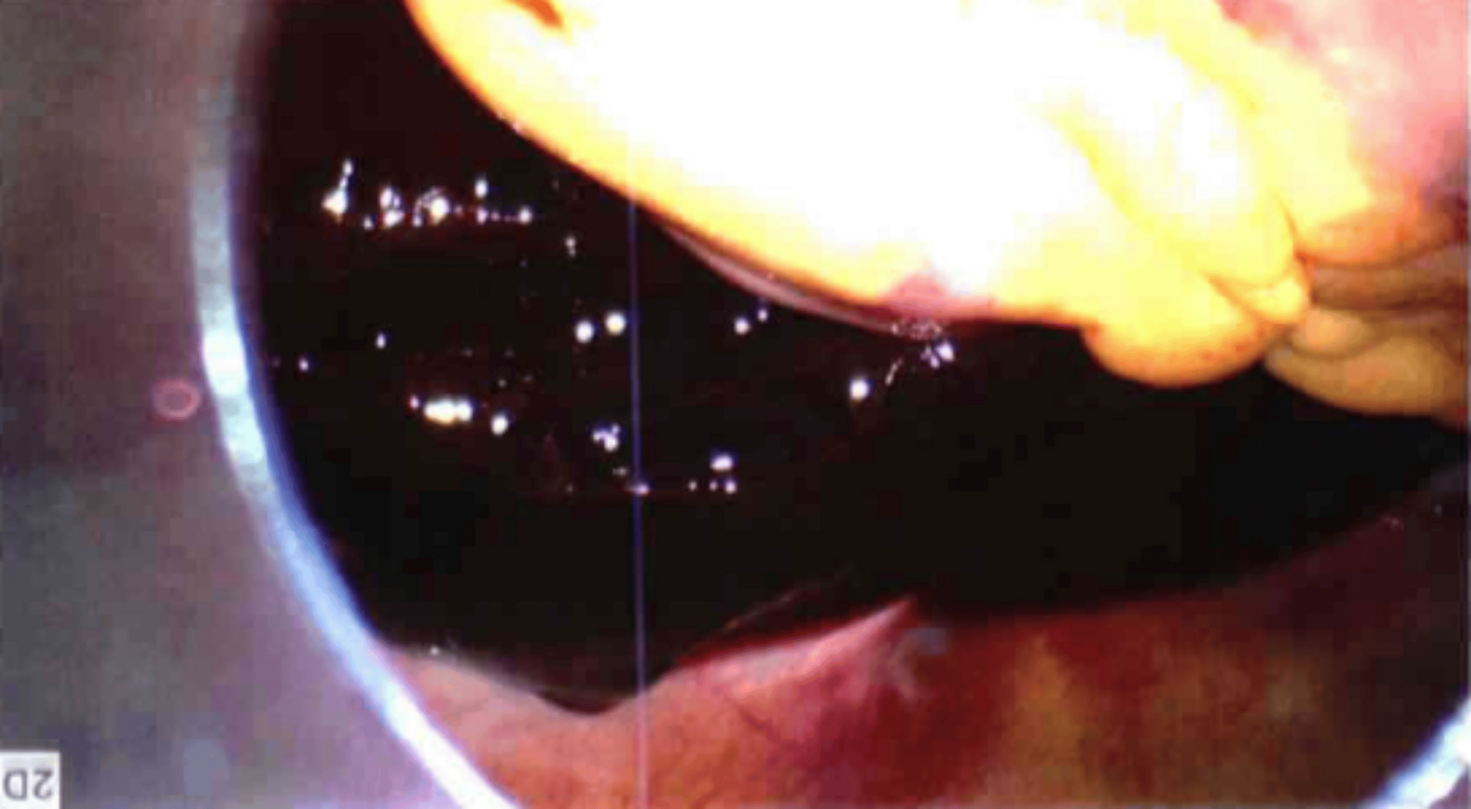
Laparoscopy showing a ruptured corpus luteum.

She underwent a second diagnostic laparoscopy (Figure 4), which revealed a ruptured corpus luteum. Diathermy was performed, and hemoperitoneum was evacuated. Following discussion with hematology, the patient was commenced on monthly GnRH analogue injections (goserelin) for ovulation suppression, with the duration of therapy planned to depend on the response of the aplastic anemia, continuing until hematologic stability is achieved or bone marrow transplantation is completed. She was also started on combined hormone replacement therapy, Evorel, to manage induced chemical menopausal symptoms. 

## Discussion

Aplastic anemia is a rare, life-threatening hematologic condition defined by hypocellular or aplastic bone marrow and peripheral pancytopenia, typically affecting at least two of the three hematopoietic cell lines: red cells, white cells, and platelets. Most patients present with nonspecific symptoms due to cytopenia, such as fatigue, bleeding, or infection, making diagnosis clinically challenging in its early stages [1].

Among the rare but critical presentations of hematologic disorders are bleeding complications. Spontaneous intra-abdominal hemorrhage, defined as bleeding into the peritoneal cavity without trauma or iatrogenic cause, represents a medical emergency. The clinical presentation often includes sudden abdominal pain, and diagnosis is commonly guided by imaging, especially CT [2]. Among the various etiologies, including hepatic, splenic, vascular, and coagulopathic causes, rupture of an ovarian cyst is the most frequently reported source of spontaneous hemoperitoneum in young women [3].

This risk is particularly heightened in patients with aplastic anemia, who are prone to hemorrhage due to severe thrombocytopenia, which impairs platelet plug formation and primary hemostasis. The deficiency of platelets, combined with possible vascular fragility and marrow suppression, increases the risk of spontaneous or excessive bleeding even from minor physiological events like ovulation [1,4,5].

One such physiological event is ovarian cyst rupture, which typically occurs during the menstrual cycle and involves either a dominant follicle or a corpus luteum. While most episodes resolve spontaneously, the theca interna and corpus luteum are highly vascular structures, and their rupture can occasionally result in significant hemorrhage, particularly in women with bleeding diatheses [6,7]. In such individuals, even minor ovulatory events can lead to massive hemoperitoneum requiring urgent surgical intervention [8,9]. CT imaging typically reveals intra-abdominal fluid without evidence of trauma, and the diagnosis may be confirmed intraoperatively [3,10].

There is a well-documented association between coagulopathies, both congenital and acquired, and severe hemorrhage from ruptured ovarian cysts. Case series and reviews have identified such bleeding complications in women on anticoagulation therapy or with conditions such as hemophilia, von Willebrand disease, or thrombocytopenia [8,9].

Though rare, ovarian hemorrhage has also been reported in association with aplastic anemia. Several case reports have described corpus luteum rupture leading to hemoperitoneum in patients with previously diagnosed aplastic anemia [11-13]. However, cases in which spontaneous hemoperitoneum from a ruptured ovarian cyst serves as the initial clinical presentation of underlying AA are exceedingly uncommon [14]. In such scenarios, the acute presentation of abdominal pain and intra-abdominal bleeding may obscure the underlying hematological disorder, delaying definitive diagnosis and appropriate treatment. These cases often prompt hematological work-up and bone marrow examination, leading to the diagnosis of bone marrow failure syndromes such as aplastic anemia [14].

In women with repeated cyst-related hemorrhages, ovulation suppression through hormonal therapy, including oral contraceptive pills or GnRH analogues, is often considered to reduce recurrence risk [9].

Unlike the typical presentation of aplastic anemia, our patient showed no prior symptoms of bone marrow failure, emphasizing how aplastic anemia can progress silently until a severe bleeding event arises. Recognizing life-threatening hemorrhagic complications like spontaneous hemoperitoneum as possible initial signs of underlying aplastic anemia is essential. Early diagnosis can guide timely management, including transfusions, ovulation suppression, and urgent hematology referral for definitive treatments such as immunosuppression or stem cell transplantation.

## Conclusions

Massive spontaneous hemoperitoneum due to a hemorrhagic ovarian cyst can be life-threatening and may occur in the setting of an underlying bleeding disorder. In this case, the bleeding was the first manifestation of previously undiagnosed aplastic anemia, highlighting a rare but important potential presentation of this condition in women of childbearing age. Notably, the patient was previously well with no prior symptoms suggestive of bone marrow failure. This case underscores the importance of considering underlying hematological disorders as a possible cause when evaluating significant hemoperitoneum secondary to ovarian cyst rupture, especially in the absence of other risk factors.
